# Miss attending risk factors in gynecological prenatal care among pregnant women at risk for dual pathology

**DOI:** 10.1192/j.eurpsy.2022.814

**Published:** 2022-09-01

**Authors:** I. Caro-Cañizares, R. Carmona Camacho, C. Vidal Mariño, N. Lopez Carpintero, E. Baca Garcia

**Affiliations:** 1 Universidad a Distancia de Madrid (UDIMA), Psychology, Collado Villalba, Spain; 2 Hospital Fundación Jiménez Díaz, Psychiatry, Madrid, Spain; 3 Hospital Infanta Leonor, Obstetricia Y Ginecologia, Madrid, Spain; 4 Universidad Autonoma MAdrid, Psychiatry, Madrid, Spain

**Keywords:** dual pathology, mental health, care access barriers, Pregnancy

## Abstract

**Introduction:**

Access to adequate healthcare is the best means we have for detecting and preventing complications during pregnancy and childbirth. Identifying and preventing factors that can interfere with this access become essential (Gulliford et al., 2002). Mother dual pathology during pregnancy is a condition with severe consequences (Cosp & Ontano, 2009). However there is scarce literature regarding barriers to obstetric care among women at risk for dual pathology.

**Objectives:**

The main objective was to explore healthcare access barriers among pregnant women at risk for dual pathology.

**Methods:**

Framed in a broader research (The WOMAP project) 2014 adult pregnant women less than 26 weeks of pregnancy were screened in five hospitals in Madrid (Spain) between 2016-2019. If the screening test (AC-Ok scale) identified the presence of dual pathology during the last month, women were included in the clinical trial and assessed with a more extensive battery (compound by PHQ-9; GAD-7; PCL-5; AUDIT; DAST; and Fagerström Test) and a semi-structured interview.

**Results:**

163 women at risk for dual pathology were assessed. Of them, 152 (93,2%) referred to having attended all scheduled appointments. Socioeconomic level (0.184, p=0.024), depression (-0.174, p=0.034), post-traumatic stress symptoms (-0.214, p=0.011) and alcohol reporting (-0.259, p=0.045) were significantly correlated with attendance level.

**Conclusions:**

Women with more severe symptoms of dual pathology are at higher risk for misatending obstetrical appointments. Social criticism, even subtle or unintentional, related to dual pathology during pregnancy could be restraining these women to attend properly. Thus, care providers should pay attention to women’s mental health and alcohol abuse to prevent miss-attention.

**Disclosure:**

No significant relationships.

